# MIQE précis: Practical implementation of minimum standard guidelines for fluorescence-based quantitative real-time PCR experiments

**DOI:** 10.1186/1471-2199-11-74

**Published:** 2010-09-21

**Authors:** Stephen A Bustin, Jean-François Beaulieu, Jim Huggett, Rolf Jaggi, Frederick SB Kibenge, Pål A Olsvik, Louis C Penning, Stefan Toegel

**Affiliations:** 1 Centre for Digestive Diseases, Barts and the London School of Medicine and Dentistry, Queen Mary University of London, 3rd Floor Alexandra Wing Royal London Hospital, London, UK; 2 Canadian Institutes for Health Research Team on the Digestive Epithelium, Department of Anatomy and Cell Biology, Faculty of Medicine and Health Sciences, Université de Sherbrooke, Sherbrooke, QC, J1H 5N4, Canada; 3 Molecular and Cell Biology, LGC, Queens Rd, Teddington, Middlesex TW11 OLY, UK; 4 Department of Clinical Research, University of Bern, Murtenstrasse 35, CH-3010 Bern, Switzerland; 5 OIE Reference Laboratory for ISA, Department of Pathology and Microbiology, Atlantic Veterinary College, University of Prince Edward Island, 550 University Avenue, Charlottetown, P.E.I., C1A 4P3, Canada; 6 National Institute of Nutrition and Seafood Research, 5817 Bergen, Norway; 7 Department of Clinical Sciences of Companion Animals, Faculty of Veterinary Medicine, Utrecht University, the Netherlands; 8 Department of Pharmaceutical Technology and Biopharmaceutics, University of Vienna, Vienna, Austria

## Abstract

The conclusions of thousands of peer-reviewed publications rely on data obtained using fluorescence-based quantitative real-time PCR technology. However, the inadequate reporting of experimental detail, combined with the frequent use of flawed protocols is leading to the publication of papers that may not be technically appropriate. We take the view that this problem requires the delineation of a more transparent and comprehensive reporting policy from scientific journals. This editorial aims to provide practical guidance for the incorporation of absolute minimum standards encompassing the key assay parameters for accurate design, documentation and reporting of qPCR experiments (MIQE précis) and guidance on the publication of pure 'reference gene' articles.

## Background

Fluorescence-based quantitative real-time PCR (qPCR) is commonly regarded as a straightforward, mature technology and has become a ubiquitous mainstay of molecular biology. However, obtaining, analysing and interpreting qPCR data is not a trivial issue. The progressive relegation of qPCR materials and methods to online supplements is resulting in an increasing tendency for scientific publications to contain insufficient technical information for that work to be reproduced; frequently that information is lacking altogether. Equally disconcertingly, the modest details included with publications frequently reveal the use of flawed practices that are likely to result in the publication of erroneous conclusions. This often makes it impossible for reviewers and readers to judge a manuscript's technical adequacy, whilst impeding journal editors' ability to reach publication decisions based on its validity and coherence.

**Figure 1:**
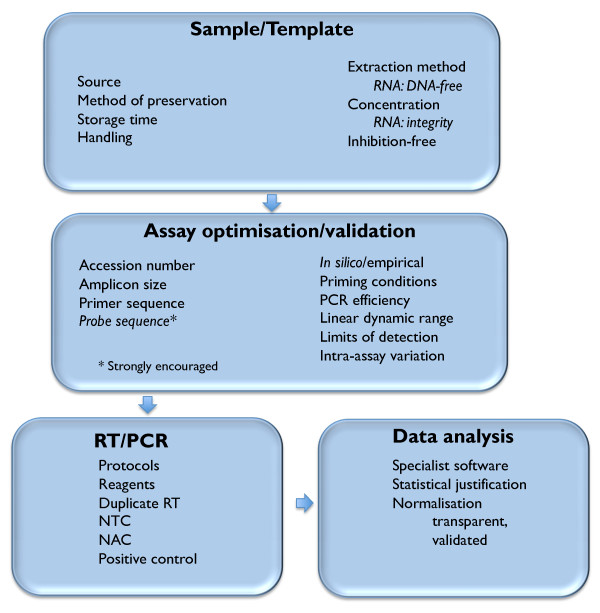
**Key criteria delineating essential technical information required for the assessment of a RT-qPCR experiment **.

A set of "MIQE" guidelines proposes a minimum standard for the provision of information for publications utilising qPCR experiments [1]. These cover every aspect of sample acquisition, assay design and validation and data analysis, to provide an ideal tool for the design of *de novo *assays and are invaluable for guiding reviewers, as well as assisting decision-making by journal editors. However, their exhaustive incorporation of numerous assay-associated details can appear daunting to non-qPCR experts and may render them too complex for the routine reporting of established assays. Consequently, we would like to propose an abridged set of guidelines for covering key parameters of every qPCR assay (MIQE précis). These cover those steps of a qPCR assay that are essential for allowing reviewers, editors and readers to evaluate the technical merits of scientific publications utilising qPCR technology (Figure 1).

### Sample/template

As sample handling affects experimental variability, it is essential to report tissue source and to provide information about conditions of storage, particularly if samples have been obtained during the course of a longitudinal study stretching over several years. Any deviations from manufacturers' protocols must be recorded. For RNA templates, the extent of residual genomic DNA contamination must be reported, ideally for each sample, or if numerous samples are being used, for a representative set of samples, by comparing quantification cycles (Cq) obtained with and without reverse transcription (RT) for each nucleic acid target. Where RNA quantities are ample for accurate RNA measurement, both quantity and quality of extracts should be recorded. Although no perfect quality assessment methods exist, measurements such as those obtained from microfluidics-based systems or 3':5'-type assays can serve as basic indicators of RNA integrity [2]. Authors should not quantitatively compare RNAs of widely dissimilar quality, e.g., RIN/RQI values of 4.5 versus 9.5, and be aware that rRNAs yielding similar RIN/RQI numbers can contain mRNAs that differ significantly in their integrity. Every sample or, in the case of very large numbers, representative samples, should also be tested for the absence of inhibitors using either an "alien" spike or a dilution series of target genes.

### Assay optimisation/validation

Database accession numbers, amplicon size and primer sequences must be reported for each target. We would recommend that probe sequences and the identities, positions, and linkages of any dyes and/or modified bases are also reported. We encourage the use of validated assays, e.g. those available from RTPrimerDB, as this helps with assay standardisation and does not require extensive validation. For newly reported assays, the use of primer design software is encouraged. Primer specificity should be validated *in silico *(BLAST specificity analysis) and empirically (ideally by DNA sequencing, but certainly using gel electrophoresis or melting profiles). Evidence for the optimisation of primer and MgCl_2 _concentration, as well as that of the annealing temperature should be provided and the PCR efficiency of each assay should be reported. This is most conveniently performed by means of calibration curves, which must be included for review, although only slopes, y intercepts, linear dynamic range and LOD need be included with the publication. A measure of intra-assay variation (repeatability) and for diagnostic assays inter-assay precision (reproducibility) between sites and different operators should also be reported. For multiplexing it is also essential to demonstrate that accurate quantification of multiple targets in a single tube is not impaired.

### Reverse transcription qPCR (RT-qPCR)

Experimental conditions and reagents must be described in detail, especially what priming method has been used for cDNA synthesis. For RT-qPCR assays total RNA concentration should be similar in every sample and we encourage replicates of the RT step to improve reliability. In addition to the DNA contamination control, which need be run only once for each sample, other important controls include:

1. ***no template controls (NTC). ***These are essential for detecting PCR contamination and must be performed with every experiment. They help establish conditions for data rejection from assays utilising SYBR Green chemistry, where primer dimers may result in positive C_q_s being recorded in the NTCs. NTCs should be the last samples to be dispensed and loaded.

2. ***positive controls. ***These are most conveniently performed as target gene-specific dilution curves and are essential if PCR measurements are of low copy numbers and where it is essential to have confidence in a negative result, for example when detecting pathogens.

3. ***no amplification controls (NAC). ***These provide valuable information when using probes, as they help monitor any probe degradation.

### Data analysis

We encourage the use of specialist software for data analysis, confidence estimation and outlier identification. The handling of such data must be specified as well as the statistical methods used to evaluate assay precision. Information relating to experimental lay-out must be supplied: we encourage the use of a sample maximisation strategy, i.e., running as many samples as possible in the same run, as opposed to a gene maximisation strategy that analyses multiple genes in the same run. This is because it minimises any technical, run-to-run variation between different samples for the comparison of gene expression levels. If not all samples can be analysed in the same run, identical samples that are tested in both runs ("inter-run calibrators" or IRCs) must be analysed. Measuring the difference in C_q _or the normalised relative quantity between the IRCs in different runs allows the calculation of a correction or calibration factor to remove run-to-run differences [3].

### Normalisation with reference genes

This is an essential component of a reliable qPCR assay:

1. mRNA data are most commonly normalised using reference genes as internal controls. Their utility must be validated experimentally for particular tissues or cell types using the specific experiment in question. Unless fully validated single reference genes are used, normalisation should be performed against multiple reference genes, chosen from a sufficient number of candidate reference genes tested from independent pathways using at least one algorithm (e.g., GeNorm). In general, the use of fewer than three reference genes is not advisable and reasons for choosing fewer must be specifically addressed.

2. For large-scale miRNA expression profiling experiments normalisation should be performed against the mean expression value of all expressed miRNAs. For small scale experiments, pilot experiments analogous to those used for mRNA reference gene selection should be used to identify suitable reference miRNAs. It is worth noting that miRNAs isolation should be carried out using specialised extraction protocols, as the efficiency of miRNA extraction is reagent-dependent and can be variable.

In recent years, biomedical journals, such as *BMC Molecular Biology*, have experienced a trend towards increased submissions of so-called 'reference gene papers', i.e., manuscripts that evaluate the stability of candidate reference genes under certain experimental conditions. While some of those studies might have provided helpful guidelines for other researchers, it is has long been undisputed that the utility of chosen reference genes must be confirmed by each research group for every experimental setup. This renders pure 'reference gene papers' pointless. Consequently, the signing editorial board members suggest that 'reference gene papers' should no longer be publishable in *BMC Molecular Biology*, except for those that:

1. Demonstrate the use of the selected reference genes for further investigations, e.g., to normalize RT-qPCR data of target genes as part of a larger study

2. Introduce and validate a novel approach for reference gene identification or normalisation, e.g., algorithms or software

3. Highlight rare cases or a standardized experimental setup of outstanding importance.

However, 'reference gene papers' that do not meet the criteria outlined here may still be suitable for peer review in other journals such as BMC Research Notes. These papers are available and will continue to be collated in a topical series (Quantitative Real Time PCR normalization and optimization).

A (RT)-qPCR checklist listing the minimum technical information required for publication in *BMC Molecular Biology *is shown in Table 1. This information can be used by the reviewers to assess the technical adequacy of the qPCR protocols, with a sentence in the published paper stating that the minimal guidelines have been adhered to. We propose that these details are included on submission as a supplemental file (please see the template included as additional file 1) for the reviewers' benefit, and, once accepted, become part of the supplementary data published online.

**Table 1:** Checklist for authors' of MIQE précis at time of manuscript submission, detailing information about individual parameters associated with each step of the RT-qPCR workflow.

Sample/Template	details
Source	If cancer, was biopsy screened for adjacent normal tissue?
Method of preservation	Liquid N2/RNAlater/formalin
Storage time (if appropriate)	If using samples >6 months old
Handling	fresh/frozen/formalin
Extraction method	TriZol/columns
RNA: DNA-free	Intron-spanning primers/no RT control
Concentration	Nanodrop/ribogreen/microfluidics
RNA: integrity	Microfluidics/3':5' assay
Inhibition-free	Method of testing

**Assay optimisation/validation**	

Accession number	RefSeq XX_1234567
Amplicon details	exon location, amplicon size
Primer sequence	even if previously published
Probe sequence*	identify LNA or other substitutions
*In silico*	BLAST/Primer-BLAST/m-fold
empirical	primer concentration/annealing temperature
Priming conditions	oligo-dT/random/combination/target-specific
PCR efficiency	dilution curve
Linear dynamic range	spanning unknown targets
Limits of detection	LOD detection/accurate quantification
Intra-assay variation	copy numbers not Cq

**RT/PCR**	

Protocols	detailed description, concentrations, volumes
Reagents	supplier, Lot number
Duplicate RT	ΔCq
NTC	Cq & melt curves
NAC	ΔCq beginning:end of qPCR
Positive control	inter-run calibrators

**Data analysis**	

Specialist software	e.g., QBAsePlus
Statistical justification	e.g., biological replicates
Transparent, validated normalisation	e.g., GeNorm summary

*Disclosure of probe sequences is strongly encouraged.

## Conclusions

It is becoming increasingly obvious that it is no longer possible to assess the technical adequacy of qPCR-based peer-reviewed publications by scrutinising either their materials and methods or online supplements sections. We propose the above guidance to uphold the quality of research published in *BMC Molecular Biology *and to help standardise the way in which qPCR-based experiments and data are reported.

## Supplementary Material

Additional file 1 **MIQE précis template for including as supplemental files in submitted manuscripts**. *Strongly encouraged. Click here for file
